# Remodeling of the Intracardiac Ganglia During the Development of Cardiovascular Autonomic Dysfunction in Type 2 Diabetes: Molecular Mechanisms and Therapeutics

**DOI:** 10.3390/ijms252212464

**Published:** 2024-11-20

**Authors:** Anthony J. Evans, Yu-Long Li

**Affiliations:** 1 Department of Emergency Medicine, University of Nebraska Medical Center, Omaha, NE 68198, USA; antevans@unmc.edu; 2 Department of Cellular & Integrative Physiology, University of Nebraska Medical Center, Omaha, NE 68198, USA

**Keywords:** type 2 diabetes mellitus, intracardiac ganglia, autonomic dysfunction, ventricular arrhythmia, sudden cardiac death

## Abstract

Type 2 diabetes mellitus (T2DM) is one of the most significant health issues worldwide, with associated healthcare costs estimated to surpass USD 1054 billion by 2045. The leading cause of death in T2DM patients is the development of cardiovascular disease (CVD). In the early stages of T2DM, patients develop cardiovascular autonomic dysfunction due to the withdrawal of cardiac parasympathetic activity. Diminished cardiac parasympathetic tone can lead to cardiac arrhythmia-related sudden cardiac death, which accounts for 50% of CVD-related deaths in T2DM patients. Regulation of cardiovascular parasympathetic activity is integrated by neural circuitry at multiple levels including afferent, central, and efferent components. Efferent control of cardiac parasympathetic autonomic tone is mediated through the activity of preganglionic parasympathetic neurons located in the cardiac extensions of the vagus nerve that signals to postganglionic parasympathetic neurons located in the intracardiac ganglia (ICG) on the heart. Postganglionic parasympathetic neurons exert local control on the heart, independent of higher brain centers, through the release of neurotransmitters, such as acetylcholine. Structural and functional alterations in cardiac parasympathetic postganglionic neurons contribute to the withdrawal of cardiac parasympathetic tone, resulting in arrhythmogenesis and sudden cardiac death. This review provides an overview of the remodeling of parasympathetic postganglionic neurons in the ICG, and potential mechanisms contributing to the withdrawal of cardiac parasympathetic tone, ventricular arrhythmogenesis, and sudden cardiac death in T2DM. Improving cardiac parasympathetic tone could be a therapeutic avenue to reduce malignant ventricular arrhythmia and sudden cardiac death, increasing both the lifespan and improving quality of life of T2DM patients.

## 1. Introduction

Diabetes is one of the most common diseases worldwide, with type 2 diabetes mellitus (T2DM) making up over 90% of all diabetes cases [1,2,3]. T2DM impacts approximately 1 in 11 adults and is becoming significantly more prevalent each year [1]. It is estimated that T2DM will impact 783 million individuals worldwide by the year 2045 with associated healthcare costs to surpass USD 1054 billion [4]. T2DM is characterized by the development of reduced insulin sensitivity and subsequent insulin resistance, ultimately leading to poor glycemic control and prolonged episodes of severe hyperglycemia [5]. The long-term lack of glycemic control leads to systemic problems, with the T2DM patient population developing many associated comorbidities including nephropathy [6], retinopathy [7], neuropathy [8], and cardiovascular disease [9]. The high incidence, paired with the severe global financial burden of diabetes, necessitates further exploration of the pathophysiological mechanisms of T2DM development in the hopes of creating more efficacious and affordable treatments for diabetes patients.

In 2021, the International Diabetes Federation reported that approximately 6.7 million adults between the ages of 20 and 79 died from diabetes or related complications [10]. The leading cause of mortality in T2DM patients is the development of cardiovascular disease (CVD) [9,11]. Adults with T2DM develop CVD approximately 14.6 years earlier than those unaffected by T2DM, and the risk of CVD increases with elevated levels of fasting blood glucose [11,12,13,14]. Diabetic patients are more likely to experience severe cardiovascular events, with T2DM patients having a 53% greater risk of myocardial infarction (MI), 58% greater risk of stroke, and 112% greater risk of developing heart failure, as compared to individuals unaffected by T2DM [11]. Over 70% of diabetic patients die from a cardiovascular-related event, with over 50% of these resulting in sudden cardiac death (SCD) [15,16,17]. SCD in T2DM patients has been linked to MI [18], with half of MI-related SCD being attributed to ventricular arrhythmias thought to be brought on by damage caused by previous cardiovascular ischemic events [15,16,19]. Ventricular tachycardia/fibrillation (VT/VF) has been reported to result in decreased stroke volume, leading to pulselessness and sudden death within one hour of development [20,21]. A recent study of over 600,000 T2DM patients showed that individuals with T2DM had an 18% greater risk of developing VT/VF [22], and ventricular arrhythmias have been reported to account for one-third of all abnormal heart rhythms recorded at the time of cardiac arrest in T2DM patients [13,21]. While glycemic control is the gold standard approach for treating diabetes, multiple studies have reported that tight glycemic control alone does not significantly reduce cardiovascular event-related deaths in T2DM patients [23,24,25]. The high prevalence of ventricular arrhythmia-related SCD in T2DM patients, and the current lack of efficacious treatments to prevent SCD, has led to an increased focus on elucidating the mechanisms responsible for ventricular arrhythmogenesis, which is crucial in the endeavor to develop new therapeutic interventions aimed at improving the prognosis and reducing mortality of SCD in T2DM.

Cardiovascular autonomic dysfunction, such as sympathetic nerve overactivation and withdrawal of parasympathetic activity, is a common complication of T2DM [26,27,28,29]. One previous study suggested that the function of the vagal nerve, the primary driver of cardiac parasympathetic activity, is severely compromised in T2DM patients [30]. Withdrawal of cardiac vagal activity develops in T2DM patients [31,32,33] and has been associated with the development of malignant ventricular arrhythmia and SCD [34,35,36,37]. Regulation of cardiac vagal activity can be integrated by the regulatory circuitry at multiple levels including vagal nerve afferent at baroreceptors, central components, and efferent components (cardiac vagal ganglia) [38]. Although structural and functional alterations in every site of the circuitry could cause attenuation of cardiac vagal activity, impairment of cardiac vagal postganglionic (CVP) parasympathetic neurons located in the intracardiac ganglia (ICG) might be an important factor for the withdrawal of cardiac vagal activity in T2DM, because (1) cardiac vagal ganglionic neurons provide local neural coordination independent of higher brain centers [39,40,41,42]; (2) acetylcholine (ACh) release from cardiac vagal neurons is blunted in T2DM patients [43]; (3) our previous study found that the cell excitability of CVP neurons was reduced due to a lower expression of voltage-gated Ca^++^ channels in T2DM rats [44]. Clinical studies and animal experiments support the notion that structural and functional remodeling of ICG neurons contributes to cardiac vagal withdrawal and the subsequent development of malignant ventricular arrhythmia in T2DM [45,46,47,48], but a comprehensive evaluation of the data has not yet been presented. Therefore, in this review, we discuss the T2DM-induced remodeling of ICG, molecular mechanisms contributing to ICG remodeling, and the potential role that the remodeling of CVP parasympathetic neurons plays in cardiovascular parasympathetic withdrawal, ventricular arrhythmogenesis, and SCD in T2DM.

## 2. The Intrinsic Cardiac Nervous System

### 2.1. Anatomy of the Cardiac Parasympathetic Nervous System and Intrinsic Cardiac Nervous System

**Figure 1:**
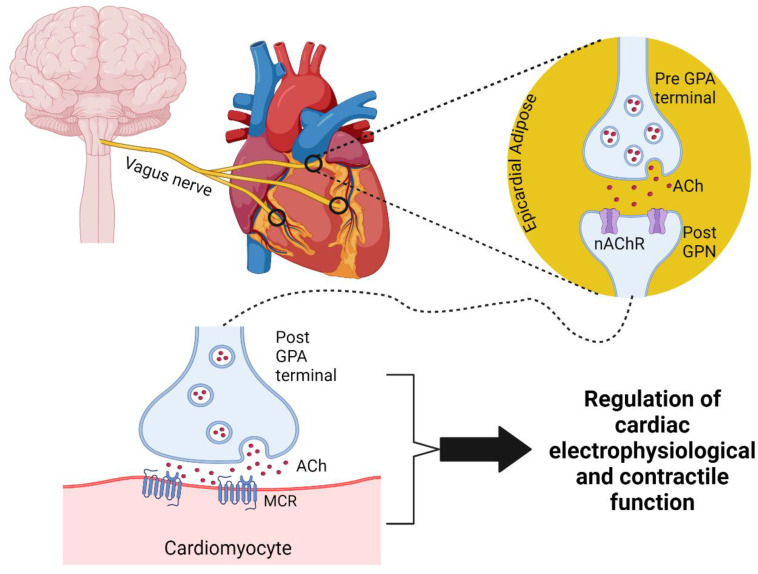
A schematic diagram of the anatomy and physiology of cardiac parasympathetic innervation of the heart. GPA: ganglionic parasympathetic axon, GPN: ganglionic parasympathetic neuron, ACh: acetylcholine, MCR: muscarinic cholinergic receptor, nAChR: nicotinic acetylcholine receptor. Figure was created in BioRender.com.

The physiological control of hemodynamic parameters such as heart rate, conduction velocity, and force of cardiac contraction is the result of a summative response of two competing divisions of the autonomic nervous system: the parasympathetic and sympathetic nervous system [49,50,51]. The parasympathetic nervous system is composed of pre- and postganglionic neurons that transmit signals to cardiac, respiratory, and endocrine organs, among others, and regulate a variety of functions [49,52,53]. Parasympathetic innervation of the heart begins with the cardiac vagal parasympathetic preganglionic neurons whose axons extend from the dorsal vagal motor nucleus (DVMN) and the nucleus ambiguus (NA) through the cardiac extension of the left and right vagal trunks and synapses onto efferent CVP neurons located in the ICG, found in the epicardial fat pads of the posterior atria, atrioventricular, and intraventricular grooves [40,54,55]. These postganglionic cholinergic neurons make up a large portion of the intrinsic cardiac nervous system (ICNS) (discussed below) that projects throughout the heart [54,56] (Figure 1).

The ICNS is the final level of neural integration controlling autonomic efferent cardiac function. The ICNS is composed of a network of ICG made up of 200–1000 neurons each [40,56]. These ICGs are connected to adjacent ganglia via interneurons creating a ganglionated plexus (GP). These GPs act as centers of integration and control for the heart [57,58]. The size, composition, and anatomical distribution of the GPs located in epicardial fat pads varies across species, with larger species (i.e., humans and pigs) having more dispersion and higher density of innervation of cells bodies as compared to smaller species (i.e., rats and mice) [56,59,60,61,62]. Anatomical studies of GPs in rat hearts report that GPs are concentrated in three distinct regions of the heart: (1) at the junction of the right superior vena cava and right atrium, close to the junction of the right cardiac vagal branch and right atrium, adjacent to the sinoatrial node (SA node); (2) at the junction of the pulmonary veins that drain into the right inferior aspect of the left atrium, adjacent to the atrioventricular node (AV node); and (3) the left superior sides of the pulmonary veins at the border of the left atrium [63,64]. A study of GPs in mice found that all GPs were clustered on the dorsal side of the heart adjacent to the pulmonary veins, and varying numbers of ganglia were found in each mouse, ranging from 13 to 23 [62]. Human GPs are concentrated in seven distinct sub-regions: the left and right coronary, the left and middle dorsal, the ventral left and ventral right atrial, and the dorsal right atrial [54,56]. These GPs singularly and/or synergistically regulate the activity of key heart structures, such as the SA node and AV node, as well as the right and left atria and ventricles [42,57]. For example, in humans, the nerves derived from the left coronary, middle dorsal, and the dorsal right atrial regions innervate the pulmonary veins [65,66]. Similarly, the GP located in the dorsal right atrial region also exhibits control over the SA node [66,67]. In rats, stimulation of the ganglia located between the superior vena cava and aorta, adjacent to the SA node, induced elongation of the P-P interval, resulting in bradycardia [63]. The regulation of the neurochemical signaling through these GPs is what controls the heart rhythm from beat to beat.

### 2.2. Neurophysiology of the Cardiac Parasympathetic Nervous System and Intracardiac Ganglia

Under physiological conditions, the parasympathetic nervous system is responsible for regulating homeostatic parameters of many tissues in the body, especially mediating the “rest-and-digest” response which keeps the body’s heart rate, metabolic rate, and respiratory rate consistent in times in which the body is safe and calm [50,68,69]. Communication from cardiac efferent preganglionic neurons to CVP neurons located in the ICG occurs primarily through the release of ACh from preganglionic neurons which binds with nicotinic acetylcholine receptors (nAChR) expressed on the membrane of the CVP neurons, but nitric oxide (NO) and vasoactive intestinal peptide (VIP) have also been reported [40,70,71]. Postsynaptic nAChR activation in CVP neurons induces cell excitation through the modulation of activity of neuronal ion channels such as nAChR, Ca^++^, K^+^, and Na^+^ voltage-gated channels [46,72,73,74,75]. Excited CVP neurons exert their effect within the ICG via the release of chemical stimuli, which in turn propagates that signal to other local circuit and efferent neurons prompting the appropriate modulation to cardiac response, named as the little brain on the heart [76,77]. Recent studies have elucidated that the ICG neurons are made up of a heterogeneous cell population of cholinergic and adrenergic neurons [59,78,79]. Using double immunostaining, Horackova et al. characterized intracardiac neurons in guinea pigs, finding that the majority (75–100%) of ICG neurons exhibited cholinergic characteristics (choline acetyltransferase; ChAT^+^), while less than 10% of ICG neurons were considered adrenergic (tyrosine hydroxylase; TH^+^) [80]. These results were further supported by Tompkins et al., showing that in mice, pigs, and humans, there were large populations of postganglionic cholinergic neurons (vesicular acetylcholine transporter; VAChT^+^) in epicardial ganglia, whereas for some neuronal cell bodies in all species stained with TH^+^, only human nerve terminals exhibited any level of TH^+^ staining [59]. There were no ICG neurons stained with neurosubstance P (SP) and calcitonin gene-related peptide (CGRP) (two sensory neuronal markers) in mice, pigs, and humans [59]. Similarly, in rats, all ICG neurons were stained with ChAT^+^. While nerve fibers within the rat ganglia stained positive for the sympathetic nerve markers TH and pituitary adenylate cyclase-activated peptide, neither of these markers were detected in ICG neuronal cell bodies [61]. Additionally, sensory neurons were not found in rat ICG [61]. While these studies support the notion that parasympathetic tone is the primary regulator of CVP neuron activity, it is possible that sympathetic and sensory nerve fibers innervate the ICG to modulate the function of the postganglionic parasympathetic neurons.

ACh released from postganglionic parasympathetic nerve terminals exerts its effect through the activation of the muscarinic cholinergic receptors (including M1–M5 subtypes); a family of G-protein coupled receptors [81]. The M cholinergic receptors exhibit a tissue specific expression, with M2 receptors being the predominant muscarinic cholinergic receptors in cardiac tissue. The M1 and M3 receptors have also been reported to be expressed in the heart, but to a much lesser extent [82,83,84]. The M2 cholinergic receptors exert chronotropic effects through multiple intracellular signaling pathways. M2 receptor activation causes the G_βγ_ subunits to dissociate from the receptor and in turn directly activate acetylcholine-sensitive potassium channels, resulting in changes in the potassium current, and ultimately inducing cell hyperpolarization [85]. The M2 receptor activation at the SA node and AV node results in hyperpolarization of the nodes, which in turn decreases cardiac conduction velocity, reduces atrial contraction force, and ultimately induces bradycardia [85,86,87,88].

Acetylcholine-activated M2 cholinergic receptors also contribute to heart homeostasis more indirectly through their impact on the levels of the second messenger cyclic AMP (cAMP). The sympathetic nervous system primarily initiates its excitatory response in cardiac tissue through stimulating cAMP production and therefore activating downstream cAMP-dependent pathways [49]. Upon ACh stimulation, the G_i_ subunit of the M2 muscarinic GPCR dissociates from the receptor and directly inhibits adenylyl cyclase, a key enzyme responsible for converting ATP into cAMP, ultimately reducing intracellular cAMP levels [89,90]. As such, activated M2 receptors act as an antagonizing force against the elevation of heart rate brought on by the sympathetic nervous system.

Alteration of the structure and/or function of the CVP parasympathetic neurons with their efferent nerves has serious implications on the homeostasis of the heart. Changes in the ICNS and associated nerves have been implicated in many cardiac pathophysiological conditions, such as atrial fibrillation, ventricular arrhythmia, and MI [91,92,93]. As such, in the following sections of this review, we elucidate the relationship between remodeling of the cardiac parasympathetic nervous system and the development of cardiovascular autonomic dysregulation in T2DM.

## 3. Remodeling of CVP Parasympathetic Neurons and Its Role in Cardiac Vagal Withdrawal, Malignant Ventricular Arrhythmia, and Sudden Cardiac Death in T2DM

Diabetic cardiac autonomic neuropathy (DCAN) is a common pathophysiology shared among T2DM patients, with 15% to 42% of T2DM patients reporting symptoms of cardiac autonomic dysregulation [94,95]. Imbalance between the sympathetic and parasympathetic inputs to the heart have previously been reported as a driver of ventricular arrhythmogenicity [35,96]. While the role of altered cardiac sympathetic activity in arrhythmia development in T2DM patients has been extensively reviewed [29,97,98], the exact mechanism leading to T2DM-induced withdrawal of cardiac parasympathetic tone has not yet been fully established. Diminished cardiac vagal parasympathetic activity is one of the first indicators of DCAN, often beginning to develop even before diabetes has been diagnosed [99,100]. As of now, a systematic evaluation of the impact of T2DM-induced CVP parasympathetic neuron remodeling in the ICG on the development of malignant ventricular arrhythmia and SCD has not yet been performed. Previous studies, including our work, have reported structural and functional changes in CVP parasympathetic neurons in T2DM models that contribute to ventricular arrhythmogenesis and SCD [45,46,48].

### 3.1. Cardiac Parasympathetic Withdrawal in T2DM

DCAN can develop as a result of dysregulation of the cardiac sympathetic branch, cardiac parasympathetic branch, or simultaneous dysregulation of both branches [101]. DCAN development is characterized in three stages. Stage 1 is preclinical with patients exhibiting decreased baroreflex sensitivity and an abnormal sympathovagal balance. Stage 2 patients experience cardiac tachycardia at rest. Patients classified as stage 3 develop cardiomyopathy with left ventricular dysfunction, orthostatic hypertension, and silent myocardial ischemia [102,103,104]. One study of 165 patients diagnosed with diabetes mellitus reports that individuals with diabetes exhibit a resting vagal tone that is 40% lower than healthy individuals [105]. Withdrawal of cardiac vagal parasympathetic tone has been reported to predate cardiac sympathetic dysfunction in T2DM patients, resulting in sympathetic overactivation leading to arrhythmogenesis and SCD [106,107].

Cardiac vagal parasympathetic withdrawal in T2DM patients is diagnosed through indices, such as elevated resting heart rate (HR), reduced heart rate variability (HRV), and a diminished baroreflex response [99,100,101]. It is believed that T2DM individuals exhibit an increased HR due to unopposed sympathetic outflow with cardiac parasympathetic withdrawal [99,107]. One of the most common clinical manifestations of parasympathetic withdrawal in T2DM patients is a reduction in HRV. Reduced HRV in T2DM patients has been associated with age, HbA1c levels, hyperglycemia, serum insulin levels, as well as the development of hepatic steatosis [32,33,108]. In a study of forty T2DM patients with an average duration of diabetes of 9.5 years, Ramussen et al. found that T2DM patients experienced reduced parasympathetic activity, as measured by a reduced deep breathing expiratory/inspiratory ratio (E/I ratio), and reduced HRV, while no significant changes in sympathetic activity were observed [101]. A meta-analysis of HRV in T2DM including 25 case-controlled studies consisting of 1356 T2DM patients revealed T2DM patients showed significantly reduced HRV parameters including reduced RR intervals and lower high frequency power (one index of cardiac parasympathetic activation in HRV analysis) [109]. A similar cross-sectional study of 160 T2DM patients stratified by disease duration found that all T2DM patients with disease durations ranging from less than 5 years to greater than 10 years exhibited lower HRV, whereas no changes in HRV were observed in healthy subjects, or patients diagnosed with prediabetes [110].

The baroreflex is regulated at multiple levels including peripheral afferent nerves located at baroreceptors, a central component, and efferent components including sympathetic and parasympathetic nerves [111]. Alterations in any site of the arterial baroreflex arc can contribute to cardiac parasympathetic nervous withdrawal. Previous studies have shown that parasympathetic preganglionic neurons found in the NA extensively project via the vagus nerve to the ICG, and that baroreflex control is abrogated when domoic acid lesions in the NA blocks projections to the ICG [112,113]. Furthermore, pharmacological inhibition of DMVN neurons has been shown to lower the ventricular effective refractory period, prolong QTc intervals, and trigger ventricular tachycardia [47]. Attenuation in baroreflex sensitivity has been widely associated with T2DM [114,115,116] and linked to arrhythmogenesis and SCD [117,118]. Therefore, elucidating mechanisms driving diminished cardiac parasympathetic tone is essential in the effort to prevent ventricular arrhythmogenesis and SCD in T2DM patients.

### 3.2. Structural Remodeling of Intracardiac Ganglia in T2DM

The terminal mediator of cardiac parasympathetic tone is CVP parasympathetic neurons located in the ICG that exert cardiac control through the release of neurotransmitters, such as ACh and NO, which bind to receptors on the surface of cardiomyocytes. While alterations in neuron structure have been reported to contribute to the development of diabetic neuropathy [119], the data on the structural changes in ICG neurons as T2DM develops is limited. In a study of Goto-Kakizaki (GK) rats, a genetic model of T2DM, immunohistochemistry of AChE-stained intact hearts revealed T2DM rats exhibited a reduced total number of ICG. The loss of ganglia appeared to be regionally specific as significantly fewer ganglia were found at the heart hilum, but no difference was observed in the number of ganglia in the dorsal atrial region. In both regions, the total area of each ICG was significantly reduced [48]. Furthermore, examination of neuronal soma revealed each T2DM ICG had fewer total numbers of neuronal soma, and each soma size was smaller as compared to controls [48]. Similar results were observed in a streptozotocin (STZ)-induced rat diabetes model, with 8-week diabetic rats exhibiting reduced numbers of ICG neurons in ganglia isolated from the vena cava, aorta, posterior left, and posterior right atrium. Transverse sections of the diabetic heart showed that diabetic ICG neurons exhibited smaller diameters consistent with the results of the Batulevicius et al. study [120]. Examination of sections of the STZ-diabetic rat posterior heart wall, using electron microscopy, revealed that the degeneration of ICG dendrites, axon terminals, and myelinated axons begins during the acute phase of diabetes induction (days 3–7), and degeneration became progressively worse between 1 and 6 months post-induction [121]. A study of human diabetic cardiac ganglionic cells, isolated by autopsy, noted that neurons in cardiac ganglia obtained from patients categorized as having severe diabetes exhibited cellular contraction, cytoplasmic condensation, widening of inter-neuronal spaces, and poor Nissl staining. No neurolytic phenomena was observed in cardiac ganglia neurons isolated from non-diabetic or mildly diabetic patients [122]. While these studies in diabetic rodent models and humans seem to support the notion that T2DM induces morphological changes in cardiac ganglia neurons, further studies examining specific sympathetic and parasympathetic nerve markers in ICG could be used to more definitively demarcate the difference in the impact T2DM has on structural changes of cardiac sympathetic and parasympathetic innervation. Further investigations of the changes in structures and functions of subcellular organelles, such as the mitochondria, nucleus, and endoplasmic reticulum, could aid in the understanding of the intracellular mechanisms driving T2DM-induced structural ICG remodeling.

### 3.3. Functional Remodeling of Intracardiac Ganglia Neurons in T2DM

Neuronal function, mediated through the propagation of action potentials, is to relay messages throughout the body, over varying distances. Cardiac vagal nerve activity is decreased in T2DM, which can be attributed to reduced cardiac parasympathetic postganglionic nerve activity [45,101], and disruption in CVP parasympathetic neuron activity has been linked to ventricular arrhythmogenesis [45,123]. Studies have demonstrated that CVP parasympathetic neurons isolated from the ICG of a high-fat diet (HFD) with a low dose of STZ-induced T2DM rat hearts exhibit reduced cell excitability and lower frequency of action potentials, indicating ICG neuron dysfunction [44,45,46]. As action potentials are induced through changes in membrane potential, studies on T2DM ICG neuron dysfunction have primarily centered on T2DM-induced changes in ion channel expression and function. While various classes of ion channels are involved in the generation and propagation of action potentials, voltage-gated Ca^++^ channels have previously been shown to play a role in the rising phase of ICG neuron action potentials [124]. Upon examination of Ca^++^ currents in CVP neurons isolated from rat hearts, Liu et al. reports that T2DM CVP neurons exhibit reduced calcium currents, indicating calcium channel dysfunction [44]. Five subtypes of calcium channels (T, L, N, P/Q, and R) have been characterized in the central and peripheral nervous system, and the pharmacological and biophysical properties of all calcium channels are determined by the alpha subunit [125]. Expression of three major gene families determine the calcium channel subtype: Ca_v_1 family (Ca_v_1.1, Ca_v_1.2, and Ca_v_1.3) encoding L-type calcium channels, Ca_v_2 family encodes P/Q (Ca_v_2.1), N (Ca_v_2.2) and R (Ca_v_2.3)-type calcium channels, and Ca_v_3 encodes T-type calcium channels [125]. It has been previously demonstrated that low-threshold calcium channels (T-type) are not expressed in rat ICG [124]. Immunofluorescence staining and RT-qPCR of CVP neurons isolated from rat hearts found that the Ca_v_1 and Ca_v_2 gene families are expressed in CVP neurons, and T2DM specifically reduces the expression of both the Ca_v_2.2-encoding transcript and protein, resulting in diminished N-type calcium channel function and reduced cell excitability [44]. Inhibition of Ca_v_2.2 expression in T2DM rat CVP neurons was induced through elevated levels of hydrogen peroxide (H_2_O_2_) (a type of reactive oxygen species, ROS) [73]. In vivo reestablishment of catalase expression, a H_2_O_2_ scavenger, via microinjection of an adenovirus carrying the catalase gene sequence into the ICG, decreased H_2_O_2_ levels, and improved CVP neuronal excitability, indicating that ROS is a driver of CVP neuronal dysfunction and ventricular arrhythmias in T2DM rats [73].

Ligand-gated nAChR, which have previously been reported to be expressed in CVP neurons, play a role in ICG neuronal signaling [126,127]. Cardiac vagal preganglionic neurons extend from the brain (DVMN and NA) and communicate with CVP parasympathetic neurons in ICG through the release of ACh. nAChR receptors on the surface of CVP neurons are activated and subsequently regulate cell excitability [128]. Liu et al. demonstrated that CVP neurons from HFD with a low dose of STZ-induced T2DM rats exhibit reduced nAChR currents, and T2DM rats exhibited reduced HRV in response to vagal nerve stimulation [46]. The exact mechanism driving reduced nAChR currents in T2DM rat ICG has not yet been established, as immunofluorescence staining showed no difference in the expression of the subunits of nAChR (α3 and β4) that control receptor activation [46]. Potassium voltage-gated channels have also been reported to influence CVP neuron cell excitability [75], but the impact of T2DM on K^+^ channel activity in CVP neurons has not been established. As T2DM has been shown to promote ATP-sensitive K^+^ channel dysfunction in β-islet cells [129], exploring T2DM-induced changes to K^+^ channel function should be further explored.

While the data predominantly support the notion that CVP neuron dysfunction develops in T2DM and contributes to the development of malignant ventricular arrhythmia, it should be noted that a recent study by Jungen et al. examined CVP neurons in the *db*/*db* genetic mouse model of T2DM and found that single stimulation of presynaptic input to CVP neurons results in a suprathreshold excitatory postsynaptic potential, which induced action potentials, indicating no dysfunction in cardiac postganglionic parasympathetic signaling [97]. Furthermore, immunofluorescence staining of VAChT, a cholinergic nerve marker, in ICG showed no changes in the number of parasympathetic ICG neurons in T2DM mice [97]. The conflicting results of this as compared to other studies could be attributed to different T2DM models. As *db*/*db* mice are diabetic due to homozygous inheritance of defective leptin receptor alleles, the molecular pathogenesis that leads to the diabetic state (hyperglycemia, hyperlipidemia etc.) may be different than an HFD with a low dose of STZ-induced diabetic model, where β-islet cell dysfunction is chemically induced. Furthermore, the interspecies variability of cardiac neuroanatomy may play a role. The mouse vagus nerve is composed of a single fascicle as compared to the multiple fascicles found in humans [130], and the distribution of cardiac GPs in mice, rat, and human hearts varies (see Section 2.1). As anatomical remodeling of rat ICG in response to T2DM shows regional specificity, with reductions in GP density at the heart hilum but not at the dorsal atrial region, while mice GPs are only clustered at the dorsal side of the heart adjacent to the pulmonary veins, it is possible that mice CVP neurons may be more resistant to structural and functional remodeling to preserve cardiac autonomic function, as they lack compensatory GPs located in other heart regions [48,62].

### 3.4. Changes in M2 Muscarinic Receptors in T2DM Rat Hearts

One potential consequence of reduced ICG excitation is the attenuated release of ACh from CVP parasympathetic nerve terminals. Reduced activation of ACh-sensitive M2 muscarinic receptors on cardiomyocytes can also cause dysregulated intracardiac signaling, resulting in cardiac arrhythmogenesis [87,131,132]. We have recently identified attenuated ACh release from CVP nerve terminals in T2DM rat hearts [133]. One previous study also found that the atria of human patients with late-stage diabetic complications exhibit reduced ACh levels [43]. While it is also possible that dysfunction in M2 receptor signaling could contribute to T2DM-induced cardiac dysfunction, a previous study reported that T2DM rat hearts exhibited no significant difference in M2 receptor expression as compared to healthy controls [134]. Furthermore, our data from isolated Langendorff-perfused hearts demonstrated that there is no significant difference on the sensitivity of the heart to exogenous ACh treatment in sham and T2DM rats, which indicates that changes of the heart (including M2 receptors) might not be involved in the impairment of cardiac vagal activation in T2DM [46]. While T2DM-induced changes in intracellular signaling pathways in cardiomyocytes is outside of the scope of this review, it should be noted that T2DM-induced changes in the structure or function of the myocardium can also be a contributor to ventricular arrhythmia development [98,135].

## 4. Cellular and Molecular Mechanisms Contributing to T2DM-Induced Cardiovascular Parasympathetic Withdrawal and Ventricular Arrhythmogenesis

### 4.1. Insulin Resistance, Glucose Toxicity, and Oxidative Stress

Insulin resistance is a common pathophysiology in T2DM patients, which has been associated with the development of cardiac autonomic dysfunction [136]. Insulin resistance occurs when cells experience diminished sensitivity to insulin, resulting in prolonged episodes of hyperglycemia [137,138]. While glucose uptake is an essential component for maintaining neuronal homeostasis, the prolonged exposure to elevated glucose levels induces glucotoxicity [139,140,141]. Glucotoxicity alters neuronal structure and function through multiple mechanisms, including an increase in the activity of the polyol/sorbitol pathway [142,143,144] and the production of advanced glycation end products (AGE) [145,146,147], which contribute to the accumulation of ROS and induce inflammation [148,149,150,151].

The polyol/sorbitol pathway has been extensively studied as a link between hyperglycemia and neuropathy in T2DM patients [144,152,153]. In physiological conditions, glucose is converted to glucose-6-phosphate and subsequently shuttled into glycolysis. When glucose levels are in excess, glucose is reduced to sorbitol, and NADPH is concurrently oxidized to NADP^+^ through the activity of aldolase reductase [142,154]. Sorbitol is then subsequently oxidized to fructose and contributes to the production of AGE (see below) [155]. Elevation of sorbitol levels has been associated with increased intracellular osmotic stress [156]. Furthermore, when aldolase reductase uses NADPH as a cofactor in the conversion of glucose to sorbitol, the intracellular NADPH pool becomes depleted. A reduced NADPH pool results in the decreased levels of reduced glutathione (GSH) [157], an essential substrate for glutathione peroxidase. Glutathione peroxidase converts H_2_O_2_ to water while concurrently oxidizing glutathione. Diminished glutathione peroxidase activity results in increased intracellular levels of ROS (such as H_2_O_2_) [142,154,157]. Many tissues in diabetic patients have been reported to experience excessive oxidative stress including the retina, kidney, as well as neurons in the central and peripheral nervous system [158,159,160,161]. Whyte et al. showed that increased levels of ROS in CVP neurons altered potassium and calcium currents to reduce neuron cell excitability [143]. The role of ROS in neuronal dysfunction was further supported by some previous studies that demonstrated that diabetic rats treated with an aldolase reductase inhibitor showed normal glutathione peroxidase activity, and that reducing oxidative stress attenuated diabetic nerve conduction deficiency and blood flow issues [162,163,164]. Campanucci et al. reported that ROS depress autonomic ganglion synaptic transmission through oxidizing the α3 subunit of nAChR, which contributes to the increased risk of fatal arrhythmia development [165]. Our studies have shown that CVP neurons isolated from HFD-low-dose STZ-induced T2DM rats exhibit elevated levels of H_2_O_2_, lower expression of the H_2_O_2_ scavenger catalase, and reduced cell excitability [44,73]. Furthermore, diminished CVP neuronal excitability has been associated with the development of MI-evoked ventricular arrhythmia [45]. Scavenging of H_2_O_2_ via overexpression of exogenous catalase in T2DM rat ICG decreased H_2_O_2_ levels and improved CVP neuronal excitability [73]. Decreased CVP neuron excitability has been linked to H_2_O_2_-induced inhibition of Ca_v_2.2 expression, resulting in diminished calcium currents in CVP neurons [44]. A recent study in NG108-15 cholinergic cells found that H_2_O_2_ induced expression of the repressor element 1-silencing transcription factor (REST) and reduced Ca_v_2.2 expression [166]. Furthermore, in vivo shRNA-mediated inhibition of REST expression in CVP neurons in T2DM rat hearts resulted in increased Ca_v_2.2 expression and improved cardiac vagal function [166].

Prolonged exposure to elevated glucose levels in diabetic patients can result in the spontaneous, non-enzymatic glycation of both intracellular and extracellular proteins [167]. During the glycation reaction, the carbonyl group of a reducing sugar, such as glucose, reacts with free amino groups on the side chains of amino acids, such as lysine, to form an unstable Schiff base. Through a subsequent series of molecular rearrangements, the Schiff base is converted to create a stable glycated protein identified as an advanced glycation end product (AGE) [145]. The pathological development of the AGE has been associated with multiple diabetes-associated pathophysiologies, such as atherosclerosis, as well as both diabetic peripheral and autonomic neuropathies [167,168,169]. Recent studies have reported that AGE accumulation in skin biopsies of T2DM patients occurs prior to and is correlated with the subsequent development of cardiac parasympathetic and sympathetic dysfunction [170,171]. Furthermore, inhibition of AGE formation via administration of aminoguanidine has been shown to attenuate atrial and ventricular tachyarrhythmic susceptibility in Langendorff perfused type 1 diabetic rat hearts [146]. AGE accumulation may impact neuronal function through both the alteration of the extracellular matrix structure, and induction of intracellular signaling pathways [147,172,173]. Glycation of extracellular matrix proteins, laminin, and collagen type IV have been shown to inhibit axonal regeneration in in vitro studies of dorsal root ganglion (DRG) neurons (sensory neurons) [147]. Furthermore, in STZ-induced diabetic mice, DRG neurons exhibited an attenuated ability to bind extracellular matrix proteins [174]. This could be attributed to glycation-induced structural changes in ECM binding proteins, such as integrins, which result in a diminished ability to crosslink to the ECM [175,176]. Similarly, Luo et al. reported that DRG neurons from STZ-induced diabetic mice grown on glycated ECM substrates presented changes in neuronal morphology, low neurite production, and a decreased survival rate of neurons [177]. Another possible explanation for AGE-induced changes in neuronal morphology and function is aberrant cross-talk between neurons and their associated glial cells. Several studies have shown that increased AGE levels induce apoptosis in Schwann cells, leading to demyelination in associated peripheral nerves [178,179]. Furthermore, glycation of neurofilament and tubulin may interfere with axonal transport and contribute to nerve fiber degeneration [180,181].

The AGE accumulation can also impact neuronal function through the activation of transmembrane bound receptors to advanced glycation end products (RAGEs). Binding of the AGE to transmembrane bound RAGE has been implicated in the activation of multiple signaling pathways, including Janus kinase, MAPK, and rho-GTPase [172,173,182,183]. A significant consequence of activated RAGE signaling is the enhanced production of ROS. A study of cultured primary DRG neurons reported that activation of RAGE resulted in increased phosphatidylinositol-3 kinase activity, and the latter facilitated elevated ROS production, caspase-3 activation, and increased degradation of nuclear DNA [151]. Similar inductions of ROS accumulation and caspase-3 activation were observed in cultured SH-SY5Y neuroblastoma cells treated with high glucose [150]. RAGE activation has also been shown to increase NADPH oxidase activity, resulting in excessive ROS production [184,185]. Elevations in ROS levels produced via either the polyol or AGE pathways have implications on cardiac autonomic dysfunction as increased oxidative stress has been associated with diminished cardiac parasympathetic activity in T2DM patients [104,186,187]. While the direct impact of the AGE on CVP neurons in the ICG have not yet been explored, as communication between CVP neurons and cardiomyocytes is essential for proper cardiac vagal signaling [54], and ROS have been shown to impact CVP neuronal cell excitability [44,45], the impact of the AGE-induced changes in cardiac postganglionic parasympathetic nerve terminals should most certainly be defined.

### 4.2. Lipotoxicity and Leptin Resistance

As CVP neurons are embedded in epicardial adipose tissue (EAT) [76], the cross-talk between adipose and neurons in the ICG may play a role in CVP neuronal dysfunction and ventricular arrhythmogenesis in T2DM. Individuals with T2DM exhibit a higher density of EAT, which has been linked to the development and progression of CVD [188,189,190,191]. Increased EAT thickness has also been associated with an increased risk of developing ventricular tachycardia [192,193]. Physiologically, EAT functions in lipid storage and energy homeostasis. It also has the ability to synthesize and release free fatty acids (FFAs) [194]. Under pathological conditions, such as T2DM, EAT-secreted adipokines can induce inflammatory cascades (discussed below), and EAT-released excessive FFAs can induce lipotoxicity, when adipokines/FFAs are taken up by neurons [195,196,197,198]. Increased plasma levels of lipid metabolites have been linked with diminished HRV in recently diagnosed T2DM patients, indicating that increases in circulating lipids may contribute to cardiac autonomic dysfunction [199]. Lipotoxicity occurs when cells take up excess levels of fatty acids, which can result in increased levels of lipid peroxidation and induce mitochondrial damage [200,201,202]. Compromised mitochondrial function further results in increased oxidative stress and potential apoptosis [203,204,205]. A recent study has shown that HFD-induced mouse obesity increases intracellular lipid accumulation in the sciatic nerve, and subsequently induces peripheral neuropathy [206]. Furthermore, peripheral neuropathy in prediabetic Zucker fatty rats has been attributed to FFA-induced oxidative and nitrosative stress [207,208]. While no direct evidence has yet been presented for the role of lipotoxicity in the development of CVP neuronal dysfunction, it is certainly possible that lipotoxicity-induced ROS elevation plays a pathogenic role in the T2DM-induced cardiovascular parasympathetic withdrawal and ventricular arrhythmogenesis.

Besides its role in FFA regulation, the EAT plays an endocrine role through the regulation and release of adipokines such as leptin, TNF-α, IL-6, and adiponectin [209]. The EAT are metabolically active tissues that, under the physiological condition, exert cardioprotective effects through regulating expression of pro-inflammatory cytokines, stimulating nitric oxide production, and reducing oxidative stress [210,211]. Greater levels of cardiac fat deposits initiate pro-inflammatory signaling, and elevated serum levels of the adipokines leptin, TNF-α, and omentin correlate with changes in HRV in T2DM patients [211,212,213,214]. The adipokine leptin exerts both pro- and anti-inflammatory effects depending on the tissues being acted on [215,216,217]. Leptin resistance is a common pathology that develops in T2DM patients, defined as patients exhibiting a reduced sensitivity to leptin [218]. One study noted that genetically leptin-resistant *db*/*db* mice, a common T2DM model, exhibited reduced HRV and baroreflex sensitivity, indicating reduced cardiac vagal tone [219]. While the exact mechanism by which leptin resistance induces cardiac vagal withdrawal in T2DM are not yet known, it may be through ROS over-production, as it has been reported that leptin resistance is associated with elevated ROS accumulation in adipocytes and the hypothalamus of T2DM mice [220,221].

### 4.3. Inflammation

**Figure 2:**
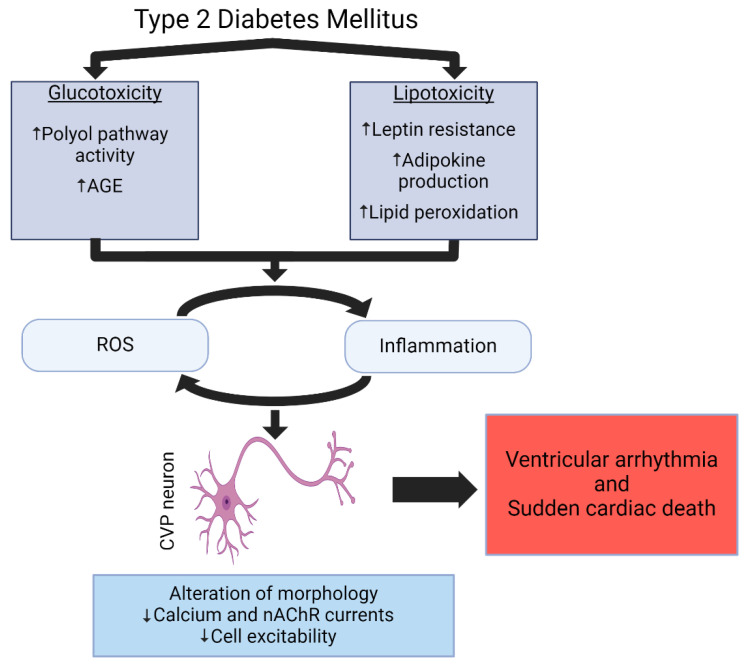
Cellular and Molecular Mechanisms Driving Cardiac Vagal Postganglionic Parasympathetic Neuronal Dysfunction in T2DM. AGE: Advanced glycation end product, ROS: reactive oxygen species, CVP: cardiac vagal postganglionic. Figure was created in BioRender.com.

The development of cardiovascular autonomic dysfunction in T2DM is multifactorial, with multiple T2DM-associated pathophysiologies, such as insulin resistance, leptin resistance, lipotoxicity, and excess EAT production, all potentially contributing to the rise in intracellular ROS and initiating inflammation (Figure 2) [114,222,223,224]. In a study of newly diagnosed diabetic patients, decreased HRV was associated with increased circulating levels of pro-inflammatory markers C-reactive protein (CRP) and IL-6 [148]. This was further supported by Herder et al. who identified that, along with CRP and IL-6, reduced cardiac vagal tone in T2DM patients was associated with elevated circulation of IL-18, soluble intercellular adhesion molecule-1, and soluble E selectin 1 [225]. A primary source of inflammatory cytokines that affect the heart is the EAT [197]. Recruitment of macrophages in hypertrophied adipose tissue, such as the EAT, release ROS in response to elevated cytokine levels [226,227,228]. Oxidative stress has been reported to activate the NFκB pathway, which drives the production and secretion of pro-inflammatory cytokines, such as IL-6 and TNF-α in the adipose tissues [149,229]. TNF-α-related inflammation leads to nerve injury, demyelination, and reduces nerve electrical conduction in diabetic peripheral neuropathies [230]. Diabetes-induced elevation of pro-inflammatory proteins may result from changes in microRNA (miRNA) expression, as changes in the expression of miRNAs such as miR-146a, miR-190a-5p, and miR-184-5p have been associated with the development of peripheral diabetic neuropathies [231]. Similarly, inhibition of miRNA miR-145-5p results in reduced levels of pro-inflammatory cytokines TNF-α and IL-6 in cultured high-glucose-treated retinal ganglionic cells [232]. While the impact for T2DM-induced changes on miRNA expression in CVP neurons has not yet been explored, defining the role of miRNA regulation of cytokine expression in the ICG is warranted. Furthermore, the efferent outflow of the vagus nerve exerts an inhibitory effect on cytokine release from immune cells [233,234], which supports the amplification of inflammatory signals in response to cardiovascular parasympathetic withdrawal. Taken together, these studies provide a strong topic that ROS and cytokine over-production could result in progressive cardiac parasympathetic withdrawal in T2DM, though this issue has yet to be definitively determined.

### 4.4. Vitamin Deficiencies

Several studies have explored the association of vitamin deficiencies and the development of cardiac autonomic dysfunction in T2DM patients. Studies of T2DM patients with 25-hydroxyvitamin D insufficiency reported that patients exhibited reduced cardiac parasympathetic nerve function as measured by the R-R variation during deep breathing and reduced HRV [235,236]. Vitamin D deficiency may be a driver for cardiac autonomic dysfunction, as rats with deprivation of vitamin D present diminished high frequency power, a reduction in the release of cardiac ACh, and a reduction in cardiac potassium channel expression [237]. Similarly, a study of 469 T2DM patients found that reduced serum levels of vitamin B12 were associated with a lower E/I ratio, a measure of parasympathetic activity [238]. Although molecular mechanisms defining the role of vitamin deficiencies in T2DM-induced cardiac autonomic dysfunction have not been established, vitamins may attenuate cardiac vagal dysfunction through ROS scavenging.

## 5. Potential Therapeutic Modalities to Treat Parasympathetic Withdrawal and Ventricular Arrhythmogenesis in T2DM Patients

### 5.1. Glycemic Control

The gold standard treatment for T2DM patients is the control of blood glucose levels to prevent hyperglycemia and subsequent oxidative stress and inflammation development. It has been well established that poor glycemic control is a driver for cardiovascular autonomic dysfunction [186,239,240,241]. While glycemic control has been shown to be beneficial in treating many diabetes-associated pathophysiologies, the impact of glycemic control on treating cardiovascular autonomic dysfunction in T2DM patients has generally been shown to provide no improvement to autonomic dysfunction [25,242]. According to the Action to Control Cardiovascular Risk in Diabetes (ACCORD) trial, T2DM patients exhibiting autonomic dysfunction had similar mortality rates when both standard and intensive treatments for glycemic control were followed [25]. Similarly, a study of a cohort of T2DM patients assigned to high-intensity interval training found improvements in HbA1c levels, but limited impact on HRV, indicating that despite improvement of blood glucose regulation, autonomic dysfunction was not improved [243]. A study of 30 T2DM patients with known cardiovascular diseases identified that T2DM patients treated with insulin and/or sufonylurea experienced high incidences of hypoglycemia, and were more susceptible to ventricular arrhythmogenesis [244]. Similar results were reported in a meta-analysis of 60 studies, in which hypoglycemic T2DM patients were found to have significantly higher risks of arrhythmic occurrence and CVD-induced death [245]. We do note that one recent study of 39 T2DM patients by Achmad et al. identified a moderately positive correlation between glycemic control and cardiac autonomic dysfunction, indicating that glycemic control may provide a slight benefit to T2DM patients experiencing cardiovascular autonomic dysfunction, though follow up studies with larger sample sizes are needed to definitely ascertain this conclusion [246]. The ambiguous role that glycemic control plays, as a treatment for cardiac autonomic dysfunction, may be due to delayed intervention. It has been reported that cardiac autonomic dysfunction begins early in the progression of T2DM, sometimes prior to T2DM diagnosis [100,110], and studies on glycemic control are usually performed on patients with established T2DM. Further studies on glycemic control in prediabetic or early stage T2DM populations may provide more insight into the potential benefits of early intervention on reversing cardiac autonomic dysfunction.

### 5.2. Pharmacological Intervention

There are two strategies for the treatment of autonomic dysfunction in T2DM patients. One is prevention of the development of dysfunction, and another is the improvement of autonomic parameters after dysfunction has developed. As the molecular mechanisms driving cardiac parasympathetic withdrawal in T2DM are diverse (Figure 2), the pharmacological therapies targeted towards improvement of cardiovascular autonomic function are varied (Table 1). One of the most common medications given to T2DM patients to control blood glucose levels is metformin. Ostropolets et al. recently reported that compared to T2DM patients treated with sulfonylurea, patients treated with metformin experienced a reduced risk for the development of ventricular arrhythmia, atrial arrhythmia, and bradycardia [247]. Similar observations were made in population-based cohort studies, in which T2DM patients in both Hong Kong and the UK treated with sulfonylurea had a significantly higher risk of ventricular arrhythmia and SCD than patients managed by metformin [248,249]. The exact molecular mechanism responsible for the potential therapeutic effect of metformin on cardiovascular autonomic function in T2DM has not yet been determined, but growing evidence suggests that it may be multifactorial, because metformin exerts both antioxidant [222,250] and anti-inflammatory actions [251,252].

Dipeptidyl peptidase-4 (DPP-4) inhibitors have recently gained attention as a potential therapeutic for treating T2DM. DPP-4 inhibitors have traditionally been used as a treatment for diabetes through the inhibition of the enzyme DPP-4, which breaks down incretins, a hormone that suppresses glucagon levels and promotes insulin production, thus regulating blood glucose levels [98]. In a recent open-label pilot study, 20 T2DM patients treated with the DPP-4 inhibitor, Teneligliptin, exhibited improvement of cardiac parasympathetic autonomic function, as measured by the heart rate responding to standing and the heart rate responding to Valsalva maneuver. In the same study, improvements in parameters for diabetic peripheral neuropathy were also observed [253]. Considering that DPP-4 inhibitors have also been shown to have adverse cardiovascular side effects such as sympathetic overactivation, and the induction of heart failure [254,255], further studies must be performed to clarify whether long-term treatment with DPP-4 inhibitors in T2DM patients is a viable therapeutic avenue.

Glucagon-like peptide-1 (GLP-1) receptor agonists have also been implicated as a potential pharmacological therapeutic treatment for T2DM-induced autonomic dysfunction, though its efficacy has recently been debated. Physiologically, GLP-1 regulates glucagon/insulin signaling pathways as an incretin hormone [256]. GLP-1 receptor agonists have been shown to impact the heart indirectly through modulation of cardiac neural circuitry [257,258]. Recently, a study of 27 newly diagnosed T2DM patients reported that treatment with the GLP-1 receptor agonist liraglutide increased heart rate and reduced HRV indicating reduced parasympathetic activity [259]. Alternatively, several studies have reported that GLP-1 receptor agonists have no impact on altering sympathovagal tone [260,261]. A recent meta-analysis studying GLP-1 receptor agonist and arrhythmogenicity in T2DM patients revealed that there was no significant association between the risk of major arrhythmias and GLP-1 receptor agonist treatment [262]. While GLP-1 receptor agonists exert positive effects on glycemic control in T2DM patients, they have not yet been shown to have any therapeutic potential for treating cardiac parasympathetic withdrawal and ventricular arrhythmogenesis.

Sodium-glucose cotransporter 2 (SGLT2) inhibitors have also been presented as a potential therapeutic for the treatment of cardiovascular autonomic dysfunction and prevention of cardiac arrhythmogenesis. SGLT2 inhibitors aid in glycemic control through inhibition of cellular glucose uptake independent of insulin action [263]. A recent study of 42 T2DM patients treated with the SGLT2 inhibitor dapagliflozin reported that dapagliflozin improved HRV and reduced the frequency of premature ventricular contractions, as compared to T2DM patients treated with other oral antidiabetics (non-SGLT2 inhibitor) [264]. Similarly, Fujiki et al. recently reported that the SGLT2 inhibitor empagliflozin reduced the number of ventricular arrhythmias in T2DM patients with an implantable cardioverter-defibrillator [265]. Alternatively, a meta-analysis of 247 T2DM patients from three independent trials reported that SGLT2 inhibitor treatment did not show any benefit for diabetes-induced cardiovascular autonomic dysfunction [266]. As such, the impact of SGLT2 inhibitors as a treatment for T2DM induced cardiac autonomic dysfunction has not yet been definitively determined.

Angiotensin-converting enzyme (ACE) inhibitors have been shown to have some therapeutic potential to treat cardiovascular autonomic dysfunction in T2DM patients. A recent study of a cohort of 18 diabetic patients treated with the ACE inhibitor Ramipril demonstrated improvement in parasympathetic tone as determined by the measurement of E/I ratio during a deep breathing test [267]. Similar improvements in cardiovascular parasympathetic tone were found when T2DM patients were treated with the ACE inhibitor quinapril for two years [268]. Alternatively, Malik et al found that one-year treatment with the ACE inhibitor trandolapril had no benefit to cardiovascular autonomic function [269]. While the studies presented here indicate ACE inhibition may improve T2DM-induced cardiovascular autonomic dysfunction, further clinical studies with larger sample sizes are necessary to definitively assess the therapeutic potential of ACE inhibitors.

**Table 1:** Summary of Pharmaceutical Therapies Targeting Cardiovascular Autonomic Dysfunction in T2DM.

Pharmaceutical Class	Citation	Treatment	Summary of Findings
Metformin	[247,248,249]	Metformin	Decreased incidence of ventricular arrhythmia and SCD
DPP-4 inhibitors	[253]	Teneligliptin	Improved heart rate in response to standing and heart rate in response to valsalva maneuver
	[254,255]	Meta-analyses including sitagliptin, alogliptin, saxaglipitin, vidaglipitin, and linaglipitin	Induced sympathetic activation and heart failure
GLP-1 receptor agonists	[259]	Liraglutide	Increased heart rate and reduced HRV
	[260]	Liraglutide and glimepiride	No change in sympathovagal tone
	[261]	Meta-analysis including exenatide and liraglutide	No change in sympathovagal tone
	[262]	Meta-analysis including lixisenatide, liraglutide, semaglutide, exenatide, albiglutide, dulaglutide, and efpeglenatide	No impact on arrhythmia development
SGLT2 inhibitors	[264]	Dapagliflozin	Improved HRV and reduced frequency of premature ventricular contractions
	[265]	Empagliflozin	Reduced occurrence of ventricular arrhythmia
	[266]	Meta-analysis including dapagliflozin and empagliflozin	No changes in cardiovascular autonomic dysfunction
ACE inhibitors	[267]	Rampiril	Improved E/I ratio during deep breathing test
	[268]	Quinapril	Improved E/I ratio and mean circular resultant during deep breathing test
	[269]	Trandoapril	No changes in cardiovascular autonomic dysfunction
Antioxidant therapy	[270]	Vitamin C	Improved HRV and decreased heart rate
	[271]	Vitamin E	Increased high power components of HRV
	[272,273]	α-Lipoic acid	No change in HRV

### 5.3. Antioxidant Therapy

As ROS accumulation has been identified as a driver for CVP neuron dysfunction resulting in the withdrawal of cardiac parasympathetic tone and ventricular arrhythmogenesis, antioxidant-based therapies are an essential therapeutic avenue that must be explored. A study by Fabiyi-Ebor et al. reported that treatment of T2DM rats with the antioxidant vitamin C inhibited T2DM-induced reduction in HRV, and decreased the heart rate, attenuating cardiac parasympathetic dysfunction [270]. Similarly, vitamin E administration improved glycated hemoglobin levels, plasma insulin levels, as well as increased the total and high frequency power components of HRV in a double-blind study of 50 T2DM patients [271]. It should be noted that not all antioxidants have been shown to be equally effective. Studies of the antioxidant α-lipoic acid (ALA) found no significant improvement in cardiovascular autonomic dysfunction in T2DM patients [272,273] despite one study that found ALA treatment significantly reduced serum levels of the pro-inflammatory proteins, CRP, IL-6, IL-8, and TNF-α [274]. Benefits of specific antioxidants in T2DM patients may be constricted by multiple factors such as dosage, metabolic breakdown, and bioavailability. Further studies are warranted to investigate specific antioxidants and their molecular mechanisms for protection against ROS production and cardiovascular vagal dysfunction in T2DM.

### 5.4. Vagal Nerve Stimulation

As cardiac vagal withdrawal is the primary driver of ventricular arrhythmogenesis in T2DM patients, vagal nerve stimulation (VNS) may have therapeutic potential for T2DM-induced cardiovascular autonomic dysfunction. Studies of VNS using implanted electrodes in pigs and canines who experienced MI found that cardiac VNS improved cardiac mechanical function and decreased the occurrence of ventricular arrhythmia [34,275,276]. Furthermore, a study of isolated innervated rabbit hearts reported that VNS corrected long QT-associated arrhythmias induced by increased sympathetic tone [277]. The beneficial impact of VNS on treating ventricular arrhythmia may be due, in part, to its ability to inhibit cytokine production. Rats subjected to 30 min of transient cardiac ischemia and VNS exhibited lower levels of inflammatory cytokines such as TNF-α and IL-6. Reductions in serum TNF-α and IL-6 were also observed in VNS-treated canines experiencing heart failure [278,279]. Similarly, VNS treatment on obese insulin-resistant rats reduced cardiac TNF-α levels and oxidative stress [280]. VNS has also been reported to reduce blood glucose levels in HFD-low-dose STZ T2DM rats [281], and improve nociceptive hypersensitivity (an indicator of diabetic peripheral neuropathy) in Zucker diabetic rats. While the beneficial impacts of VNS on cardiac parasympathetic withdrawal in T2DM has yet to be determined, the restoration of parasympathetic function remains an attractive therapeutic strategy to be explored.

## 6. Conclusions

In this review, we update information on the remodeling of cardiac postganglionic parasympathetic neurons in the ICG during T2DM development and summarized the impact of withdrawal of cardiac parasympathetic tone on ventricular arrhythmogenesis in T2DM patients. The evidence presented here supports a complex interrelationship between oxidative stress and inflammation in the progression of cardiovascular autonomic dysregulation in T2DM. No current therapy so far has proven to be definitively efficacious in the treatment of established cardiac parasympathetic dysfunction in T2DM patients. Future examinations of endogenous signaling pathways leading to oxidative stress and inflammation in cardiac postganglionic parasympathetic neurons are essential in the effort to develop targeted therapeutic interventions in the hope of preventing ventricular arrhythmia and SCD in T2DM patients.

## Data Availability

No new data were created or analyzed in this study. Data sharing is not applicable to this article.

## Abbreviations

Ach: Acetylcholine; CVD: Cardiovascular disease; CVP: Cardiac vagal postganglionic; ICG: Intracardiac ganglia; ROS: Reactive oxygen species; SCD: Sudden cardiac death; T2DM: Type 2 Diabetes Mellitus; VNS: Vagal nerve stimulation.
